# Advances of Metabolomics in Fungal Pathogen–Plant Interactions

**DOI:** 10.3390/metabo9080169

**Published:** 2019-08-15

**Authors:** Fangfang Chen, Ruijing Ma, Xiao-Lin Chen

**Affiliations:** 1 State Key Laboratory of Biocatalysis and Enzyme Engineering, Hubei Collaborative Innovation Center for Green Transformation of Bio-Resources, School of Life Sciences, Hubei University, Wuhan 430062, China; 2 The Provincial Key Laboratory of Plant Pathology of Hubei Province, College of Plant Science and Technology, Huazhong Agricultural University, Wuhan 430070, China

**Keywords:** metabolomics, metabolites, plant pathogenic fungi, fungus–plant interactions, metabolic pathway

## Abstract

Plant disease caused by fungus is one of the major threats to global food security, and understanding fungus–plant interactions is important for plant disease control. Research devoted to revealing the mechanisms of fungal pathogen–plant interactions has been conducted using genomics, transcriptomics, proteomics, and metabolomics. Metabolomics research based on mass spectrometric techniques is an important part of systems biology. In the past decade, the emerging field of metabolomics in plant pathogenic fungi has received wide attention. It not only provides a qualitative and quantitative approach for determining the pathogenesis of pathogenic fungi but also helps to elucidate the defense mechanisms of their host plants. This review focuses on the methods and progress of metabolomics research in fungal pathogen–plant interactions. In addition, the prospects and challenges of metabolomics research in plant pathogenic fungi and their hosts are addressed.

## 1. Introduction

Metabolomics originated from metabolic profiling. Researchers at the Baylor College of Pharmacy proposed and published the theory of metabolic profiling in the 1970s. With the advent and rapid development of genomics, Oliver et al. proposed the concept of the “metabolome” in 1998 [1], and many plant chemists conducted research in this area thereafter. Nicholson et al. proposed the concept of metabonomics, which was defined as “the quantitative measurement of the dynamic multiparametric metabolic response of living systems to pathophysiological stimuli or genetic modification” [2]. Since their study, a lot of research on disease diagnosis and drug screening has been conducted using metabonomics. Fiehn proposed “metabolomics” in 2001 and defined it as “a comprehensive and quantitative analysis of all metabolites in a biological system” [3]. Metabolomics focuses on all small molecule components and the fluctuations in individual cells or cell types, and is often used to study plant and microbial systems.

Interactions between fungi and their hosts represent an intriguing field which includes the interactions of fungal pathogens with plant, insect, animal, or human hosts. Among these, fungal pathogen–plant interactions are very important for agricultural production. At present, metabolomics research in both fungi and plants has been finely reviewed [4,5,6,7], but has seldom focused on the fungal pathogen–plant interactions. In the past decade, due to the importance of plant pathogenic fungi in microbial systems, metabolomics techniques have been widely used in different research fields of fungal pathogen–plant interactions, such as identifying fungi, determining infection mechanisms, and detecting interactions with the host. Metabolomics is more widely used in fungus-infected plants to understand plant defense mechanisms. Up to now, metabolomics analyses of fungal pathogen–plant interactions have mainly been performed between several fungi and their host plants, especially between *Fusarium graminearum*, *Magnaporthe oryzae*, *Ustilago maydis*, *Rhizoctonia solani*, *Botrytis cinerea*, *Sclerotinia sclerotiorum*, and their hosts. In this review, we summarize the major improvements in analytical platforms and the recent advancements in metabolomics research of fungal pathogen–plant interactions, aiming to further promote the application of metabolomics in plant pathogenic fungi research, which can help us to understand the pathogenesis of pathogenic fungi and plant defense mechanisms and eventually help us to develop new control strategies for fungal diseases.

## 2. Metabolomics Methods for Fungal Pathogen–Plant Interactions

**Figure 1:**
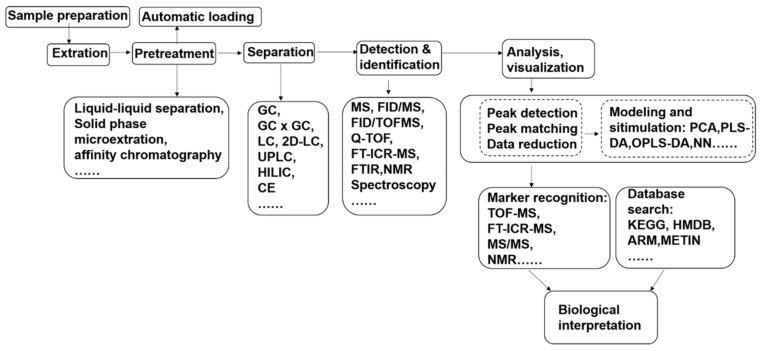
Metabolomics analysis flow for fungal pathogen–plant interaction research. PCA, principal component analysis; HCA, hierarchical cluster analysis; PLS-DA, partial least squares discriminant analysis; OPLS-DA, orthogonal partial least squares discriminant analysis; MUDA, multiple univariate data analysis; LDA, linear discriminant analysis; NN, neural networks; HMDB: human metabolome database; KEGG, Kyoto encyclopedia of genes and genomes.

Techniques in metabolomics research are still under rapid development. Hence, methodologies are constantly evolving with the expansion of the application scope [8]. Methods for experimental design, sample preparation, data acquisition, data processing, and biological interpretation relevant to metabolomics of fungal pathogen–plant interactions are described in the following sections, and Figure 1 shows the metabolomics analysis flow for fungal pathogen–plant interaction research.

### 2.1. Experimental Design

In order to obtain meaningful data, metabolomics research requires careful experimental design, in which the time, type, and groups for sample collection should be carefully considered. In order to obtain high-quality information, the problems to be solved must first be determined and appropriate research parameters and reliable experimental techniques should be selected. Based on statistics, there are many experimental design methods, including orthogonal design, single- and multiple-factor design, regression design, and central combination design. To statistically reflect the validity of the experimental data, it is necessary to consider how many samples should be selected and how many metabolites should be detected [9]. According to statistical principles, 30 cases need to be counted, and 20 cases need to be statistically significant. Apart from special situations, such as valuable classic cases, in which samples are difficult to obtain, a sample number of less than five will lead to inaccurate statistical results [10]. The smaller the sample number is, the larger the sampling error will be. If the sample number is too small, the difference can be repeated, the test efficiency will be low, and the influence of accidental factors cannot be ruled out, resulting in poor scientificity and authenticity. In contrast, if the sample number is too large, it will be difficult to strictly control the test conditions, wasting manpower, material resources, and time. 

### 2.2. Sample Preparation

Metabolomics is designed to analyze all of the information in a metabolome after stimulation or disturbance in a biological system. Although it is currently possible to analyze the levels of all metabolites, from a systematic point of view, multiple factors must be considered when collecting samples, including the source of the samples, their growth conditions, genetic information, the sampling time, the sampling interval, and the control settings. In short, to ensure the repeatability of microbial growth under constant culture conditions, the selected samples should be representative of the study subject without interfering with the research purpose. 

When the external environment changes, small molecular metabolites in the organism will also undergo rapid changes. Appropriate sample collection and preparation steps, including rapid sampling, quenching, and extraction of metabolites, are thus highly necessary. It is generally necessary to quickly freeze the samples after collection and store them in an environment below −60 °C until extraction to ensure the stability of the metabolites in the organism. 

Metabolic quenching is a key step for obtaining biological samples. Due to the activities of enzymes in the body, metabolites are degraded easily and more rapidly than mRNAs and proteins. In order to reduce metabolite degradation, certain measures must be taken to inhibit the activity of degrading enzymes, such as immediate filtration with liquid nitrogen, ultrafiltration or treatment with acids, grinding with liquid nitrogen, and dilution with pre-cooled methanol solution followed by fast centrifugation [11,12].

The extraction of metabolites is an important step in sample preparation. At present, the commonly used methods for extracting metabolites include the use of cold methanol, hot methanol, and a chloroform–methanol mixture, combined with auxiliary treatments such as ultrasonic crushing, glass ball milling, circulating freeze–thaw, and microwaving. However, the diversity of metabolites will lead to different solubilities, and it is often difficult to extract all metabolites with one single extraction method. It is thus necessary for researchers to choose different extraction methods according to the purpose of the experiment to ensure sufficient extraction of all metabolites and avoid changes in the properties of metabolites.

### 2.3. Data Collection

The separation, detection, and identification of metabolites are the core parts of metabolomics research. Gas or liquid chromatography-mass spectrometry (GC-MS or LC-MS), Fourier transform infrared spectroscopy (FTIR), and nuclear magnetic resonance (NMR) are the three main platforms for metabolomics research. In addition, there are other separation methods such as capillary electrophoresis and electrochemical detection.

The core idea of MS analysis is to ionize an isolated compound to determine the content of specific ions, which is the basis for the qualitative analysis of the compound’s properties. GC-MS analysis can simultaneously measure hundreds of chemically different compounds, including organic acids, most amino acids, sugars, sugar alcohols, aromatic amines and fatty acids, for the analysis of volatile and intermediate compounds with the advantage of high separation efficiency and a reproducible retention time [9]. The advantages of GC-MS analysis include high separation efficiency and good reproducibility, but many compounds containing polar groups require pre-column derivatization to achieve good separation [13]. The greatest advantage of GC-MS is that this method can use standard libraries for structural identification, and a large number of libraries can be retrieved [14]. LC-MS and multistage LC-MS (LC-MS^n^) can detect compounds that do not volatilize easily, thermally labile compounds, polar compounds, and macromolecular metabolites. The development of modern ion trap multistage mass spectrometry has absolute advantages in the qualitative analysis of compounds and the acquisition of structural information [15]. Capillary electrophoresis-mass spectrometry (CE-MS) has the advantages of rapid analysis, fewer required samples, low reagent consumption, and a relatively low cost. It can detect polar or charged metabolites, such as inorganic ions, organic acids, amino acids, vitamins, nucleotides and nucleosides, thiols, carbohydrates, and peptides [16]. 

With the development of metabolomics analysis technology, high-sensitivity and high-resolution MS detectors with high-efficiency separation chromatogram matching have been developed to isolate and identify biomolecules. Such detectors include two-dimensional gas chromatography with the time of flight mass spectrometer (GC×GC-TOF-MS), triple quadrupole mass spectrometry (QQQ-MS), matrix-assisted laser desorption ionization mass spectrometry (MALDI-MS), quadrupole rod tandem time-of-flight mass spectrometry (Q-TOF-MS), hydrophilic interaction liquid chromatography mass spectrometry (HILIC-MS), ion-pair-LC coupled to electrospray-ionization mass spectrometry (IP-LC-ESI-MS), MALDI-TOF-MS, and other MS detectors, and the corresponding metabolite databases appeared one after another. 

FTIR is used to determine the infrared absorption frequency and intensity of the experimental sample to identify each component, and it is mainly applied to determine the functional groups of the components in the sample and the vibration of the high polarity bonds. Besides, FTIR also has some disadvantages such as the inability to distinguish isomers, the effects of component fragments and complex ions on the analysis, and the inability to quantitatively analyze ion suppression [17].

NMR is a non-destructive and high-throughput detection technique, which is based on the magnetic properties of a nucleus with spin properties that absorbs radio frequency radiation and generates energy level transitions under the action of a nuclear external magnetic field. NMR utilizes the rich information from all small molecule metabolites in the organism provided by the NMR spectrum of biological fluids and determines the complete metabolic map of related organisms through multivariate statistical analysis and pattern recognition processing. Currently used NMR techniques include the hydrogen spectrum (^1^H-NMR), carbon spectrum (^13^C-NMR), and phosphorus spectrum (^31^P-NMR), among which ^1^H-NMR is the most widely used. The advantage of NMR over MS is that the preparation is simple, and the structure of the substance can be easily identified. In addition, the signal intensity on the spectrum is directly related to the concentration of the metabolite being detected so that the metabolite can be accurately quantified. However, the sensitivity of NMR is low, and it is difficult to simultaneously detect metabolites with large concentration differences in biological systems, which hinders its application in fungal metabolomics. In order to improve the sensitivity of NMR, the magic angle rotation NMR technique [18] and high resolution NMR were developed.

### 2.4. Data Processing and Analysis

After the chromatographic separation of biological metabolites, a large amount of spectral and multivariate data are generated [19]. Each signal peak of the spectrum contains qualitative and quantitative information about the various substances in the metabolite. Hence, it is necessary to use statistics and chemometrics for analysis. Initially, the raw data require preprocessing, including baseline correction, feature detection, noise filtering, peak extraction, peak alignment, deconvolution, and normalization to eliminate interference factors. These processes can be implemented using software, such as MetAlign [20], MZmine [21], XCMS [22,23,24], METIDEA [25], AMDIS (https://chemdata.nist.gov/dokuwiki/doku.php?id=chemdata:amdis), and MSFACTS [26]. Many instrument manufacturers have also developed their own proprietary software such as MarkerLynx (Waters, Milford, MA, USA), AnalyzerPro (SpectralWorks, Runcorn, Cheshire, UK), Progenesis QI (Waters, Milford, MA, USA), MetAlign (20), MassProfiler (Agilent Technologies, Santa Clara, CA, USA), ChromsTof (Leco, St. Joseph, MI, USA), MarkerView (Thermo Fisher Scientific, Waltham, MA, USA), and SIEVE (Thermo Fisher Scientific, Waltham, MA, USA).

Pre-processed data require multivariate statistical analysis and bioinformatics analysis [27,28], including unsupervised and supervised analyses. Unsupervised analyses include the principal component analysis (PCA) and hierarchical cluster analysis (HCA). If the differences between the sample groups are too small or the differences within the groups are too large, it is difficult to determine the differences between groups [29,30]. Supervised analyses include the partial least-squares discriminant analysis (PLS-DA), the orthogonal partial least-squares discriminant analysis (OPLS-DA), the multiple univariate data analysis (MUDA), the linear discriminant analysis (LDA), and neural networks (NN) [29,30,31,32]. These multivariate statistical analyses can help researchers to obtain potentially effective information and find biomarkers and metabolic pathways.

Metabolomics analysis requires the use of various metabolic pathways and biochemical databases [27,28]. At present, there is no well-established metabolomics database similar to those available for genomics and proteomics. Establishment of a microbial metabolomics database will accelerate the identification of compounds and species [33]. Some biochemical databases can be used for metabolic pathway analysis and structural identification of unknown metabolites. Table 1 lists the databases related to metabolomics and microbial metabolomics research for reference. An ideal metabolomics database, such as the human metabolomics database (http://www.hmdb.ca), should include the metabolome information of the organism and its quantitative data. Some public data, such as the Pubmed compound library and the ChemSpider database (Table 1), which are available for online retrieval, are also useful for identifying metabolites in various biological samples. In addition, some research institutes have also established databases of metabolites in their research foci.

## 3. Research Progress and Application of Metabolomics in Fungal Pathogen–Plant Interactions

Plant pathogenic fungi can cause a serious reduction in the crop yield and affect the quality of agricultural products [34,35,36,37]. Revealing the infection mechanisms of the plant pathogenic fungi can help us to develop novel strategies to control fungal diseases. In particular, metabolomics could provide targets for the development of new fungicides. At present, metabolomics technology is widely used in the field of plant pathogenic fungi research. Metabolomics research of plant pathogenic fungi focuses on the functions of metabolites and metabolic pathways during fungal development, pathogenesis, and interactions with plants. Metabolomics can be used to detect normal genetic development and the changes in metabolome characteristics caused by host stimulation [38], which reflects the phenotypic changes of fungi from a global point of view. Metabolomics can also be used to obtain small molecular metabolites produced by plants upon infection of pathogenic fungi [39,40]. In order to study the plant immunity, elicitors derived from the plant pathogenic fungi are also used to treat the host plant for metabolics research. Many plant pathogenic fungi such as *Fusarium* and *Aspergillus* can produce toxins in host cells. Therefore, toxin-induced changes in the plant metabolic pathways can be also finely detected by metabolomics. Combined with a variety of omics methods and techniques, metabolomics can also help to screen for resistant varieties and assisted crop breeding [39,41,42,43,44,45].

### 3.1. Progress in Metabolomics Research for Fungal Pathogen–Plant Interactions

At present, extensive progress has been made in several fungal pathogen–plant interaction systems, including the *Fusarium graminearum*–wheat interaction, *Rhizoctonia solani*, *Magnaporthe oryzae*–rice interaction, *Ustilago maydis*–maize interaction, *Botrytis cinerea*–plant interaction, *Sclerotinia sclerotiorum*–plant interaction, *Colletotrichum*–plant interaction, and *Verticillium*–plant interaction, which will be described in the following sections (Table 2).

#### 3.1.1. *Fusarium graminearum*–Wheat Interaction 

*Fusarium* head blight (FHB) is a fungal disease caused by *Fusarium graminearum* (FG), which can cause rot in various cereal crops such as wheat, corn, and barley. FHB not only affects the crop yield but also decreases the quality of agricultural products [46]. *Fusarium* is a necrotrophic pathogen. It secretes toxins to kill plant tissues and then uses dead tissue for nutrients during infection. At present, more than 300 *Fusarium* toxins, such as the deoxynivalenol (DON) toxin, have been found, and more than 100 of them are toxic to almost all eukaryotes [46].

**Figure 2:**
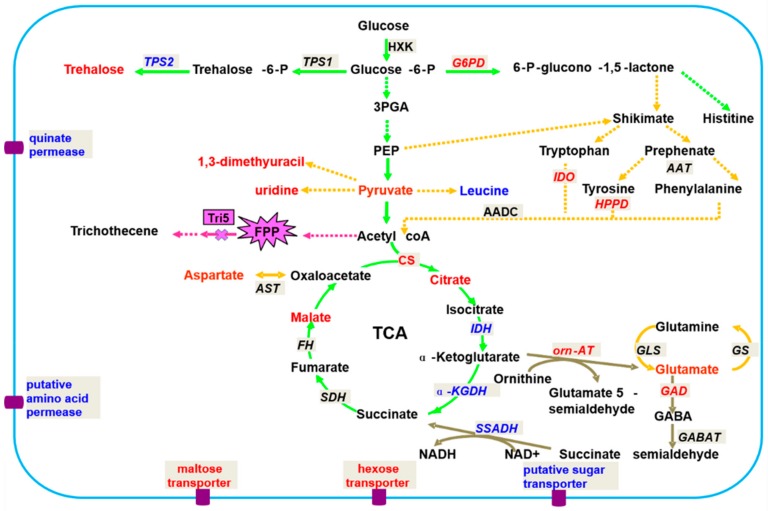
Hypothetical network of metabolism in *F. graminearum* related to 5035/*Tri5*. Red font indicates significantly up-regulated metabolites (*r* > 0.75); blue font indicates significantly up-regulated metabolites (*r* > 0.75); black font indicates metabolites detected but with low cutoff values (*r* < 0.75) or not detected in this study; the green pathway indicates C metabolism; the orange pathway indicates N-metabolism; maroon indicates the GABA shunt; the textbox with a French grey background indicates the code genes in the metabolism, the red and blue fonts indicate the significantly up/down-regulated trends, and the black font indicates no changes in the trends; significant metabolites are shown by an explosive shape. Abbreviations: 3-PGA, 3-phosphoglycerate; PEP, phosphcenolpyruvate; TCA, tricarbocylic acid cycle; FPP, farnesyl pyrophosphate.

Lowe et al. used ^1^H NMR and GC-MS to study the differences in metabolites among four different *Fusarium* strains. The results showed that the effects of the nutrient environment on fungal metabolism are greater than those of genotypes [47]. Chen et al. also employed ^1^H NMR and GC-MS to study the differences in metabolites between *F. graminearum* 5035 and *Tri5* gene deletion. The results showed that *Tri5*^−^ deletion would lead to a normal phenotype but the toxigenic ability would be lost. The primary metabolites of *F. oxysporum* vary widely. Metabolite changes include changes in carbon, sulfur, and nitrogen fluxes; the tricarboxylic acid (TCA) cycle; gamma-amino butyric acid (GABA) bypass; the shikimate pathway, and amino acids, lipids, choline, purines, pyrimidines, and other metabolites (Figure 2). These results suggest that toxins have an effect on the physiological functions of fungi and that lipids and shikimic acid-related metabolites provide some information for studying the toxigenic mechanism of *F. graminearum*. The results provide a theoretical basis and data for the further development of new biologic agents against FHB [12]. The above research results fully demonstrate that phytopathogenic fungal metabolomics can not only identify strains through the secretion of metabolites but are also an effective tool for studying metabolic pathways and the gene functions of pathogens.

More than 100 quantitative trait loci related to FHB resistance have been found in wheat and barley using QTL mapping, indicating multiple mechanisms of FHB resistance [48,49]. The Qfhs.ndsu-3BS site is known to be involved in the process of detoxification of DON to the less toxic DON-3-*O*-glucoside (D3G) [48,49], and it also confers FHB resistance [50,51]. In barley samples infected with *Fusarium*, an increase in the DON/D3G concentration was positively correlated with the increase in several plant-related metabolites including jasmonic acid (JA), dihydro-7-hydroxyglycine, kaempferol-3-*O*-glucoside-7-*O*-rhamnoside, and 4-methoxycinnamic acid [52]. Hamzehzarghani et al. used GC-MS to study the quantitative resistance of the *F. graminearum* interaction system and tentatively identified 55 metabolites, and analyzed the metabolites that play roles in plant disease resistance. The biosynthetic pathways provide a theoretical basis for the selection of new varieties with resistance to FHB [53]. Paranidharan et al. also used GC-MS to study the resistance of wheat to *F. graminearum*. After inoculation with the *F. graminearum* and *Fusarium toxin* DON, 117 metabolites were identified by Paranidharan et al. using GC-MS [54]. Tomas and Bollina used liquid chromatography with electrospray ionization coupled with LTQ-Orbitrap mass spectrometry (LC-ESI-LTQ Orbitrap MS) to study some metabolites associated with quantitative resistance in response to *F. graminearum* infection [52,55]. In barley, compared with the susceptible lines, higher levels of flavonoids, phenylpropanoids, and metabolites of fatty acids and terpenoid pathways were found in the resistant barley lines upon infection with *Fusarium* [56]. Kumaraswamy et al. also screened barley lines against FHB and found that 161 metabolites, including linoleic acid, p-coumaric acid, and naringenin, may be associated with the lower susceptibility of barley lines [57].

The complex system consisting of disease-resistant and disease-susceptible barley as well as toxin-producing and non-toxin-producing *F. graminearum* is an ideal model for studying the metabolic response of wheat to FHB [48]. In wheat-resistant varieties, JA-Ile (jasmonic acid isoleucine) and HCAAs (hydroxycinnamic acid amide, phenol polyamine conjugate), such as acyl putrescine/mercaptoamine and wheat glutamate/mercaptoamine, showed excessive accumulation. This resistance is mainly attributed to the activation of phenylpropanoid, steroid, and fatty acid metabolic pathways; and DON detoxification of D3G [48].

#### 3.1.2. *Magnaporthe oryzae*–Rice Interaction

Rice blast caused by the filamentous ascomycete fungus *M. oryzae* (also called *M. grisea*) is the most serious fungal disease in rice worldwide, causing severe yield reductions each year and significant economic losses [58]. Jones et al. used a meta-analytical method based on GC-MS/MS, LC-MS/MS, and ^1^NMR to evaluate rice at different time points after infection by compatible (KJ201) and incompatible (KJ401) strains of *M. grisea*. There was no significant difference in the metabolic response caused by each pathogen strain at 24 h after inoculation. The greatest change was found in alanine, which was about 30 ± 9% higher in the compatible strain than in the resistant strain. Together with several other metabolites, alanine shows good correlations between the time of infiltration of the leaves by the fungus and the divergence of the metabolite profile in each interaction. The authors proposed that the production of a large amount of alanine triggered by fungi may lead to cell death, thereby promoting *M. grisea* infection [59]. *M. oryzae* also produces a variety of phytotoxic secondary metabolites, such as pyrichalasin, tenuazonic acid, and magtoxin [60]. The HPLC/MS method has been used to identify pyriculol and pyriculariol as the metabolites present after *M. oryzae* infection, but pyriculol is not necessary for causing rice damage [61]. 

Recent studies have shown that phosphorylatic and phosphatidyl glycerol (PG) are associated with the resistance of rice to *M. oryzae* [62]. When rice blast fungus infected the susceptible (ABR1) and resistant (ABR5) rice, fatty acids were found to be the most important metabolites of the antagonistic species, and electrospray ionization mass spectrometer (ESI-MS) analysis identified this substance as phospholipids (PLs). PG is the main source of jasmonic acid (JA) in the host and is reduced after the attacking of rice by *M. oryzae*. Researchers predicted that JA levels would increase, and this prediction has been validated. In the early stage of inoculation with *M. oryzae*, PG-PLs were inhibited, regardless of the presence of resistant or susceptible varieties. In the disease development stage, different phosphatidic acid PLs showed rising or decreasing trends in the resistant varieties [45]. 

**Figure 3:**
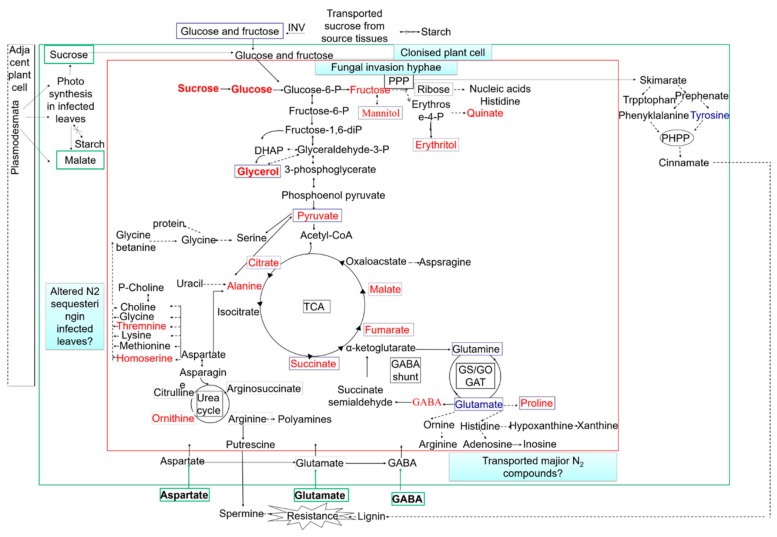
Model summarizing fungal metabolic interactions with the colonized host. INV, invertase; PPP, pentose phosphate pathway; PHPP, phenylpropanoid pathway; TCA, tricarboxylic acid cycle. The metabolites in blue boxes are fungal metabolites that are predicted to increase after 3 days. The metabolites in green boxes are the major central carbon and nitrogen compounds that are likely to be derived from the host. Red font represent the upregulation metabolites; blue font represents the downregulation metabolites. The green arrows indicate transport across cell walls. The dotted arrows indicate multiple enzymic steps.

The metabolic pathways in *Magnaporthe*-infected hosts were not fully understood until ten years ago. It was found that *M. grisea* can use a common metabolic reprogramming strategy to inhibit plant defense and colonize plant tissues during colonization in barley, rice, and *Brachypodium distachyon* [62]. Non-target metabolic profiling and GC-TOF-MS targets were detected by flow injection electrospray ionization mass spectrometry (FIE-MS) to confirm this result. In the host tissues, after pathogen infection but before the appearance of symptoms, malate and polyamine accumulated and were used to produce defensive active oxygen, and the presence of metabolites was related to the improvement of redox stress. When the infected leaf tissue showed lesions, decreased photosynthesis, the accumulation of amino acids and sugars, early transfer of the shikimate pathway to initiate the production of quinone quate as well as the accumulation of unpolymerized lignin precursors were found. In the late stage of fungal infection, when the infection hyphae rapidly expanded, the photoassimilates were conversed to mannitol and glycerol for mycelial growth [62]. The rapid proliferation of *M. grisea* hyphae in plant tissues after three days is associated with accelerated nutrient acquisition and utilization (Figure 3).

#### 3.1.3. *Ustilago maydis*–Maize Interaction 

Doehleman et al. examined changes in the transcriptome and metabolites that induce tumor formation on susceptible maize hosts [63]. *U. maydis* does not obtain more carbon nutrients through lyase and can soften cell walls during plant colonization. During tumor formation, the flavonoid pathway and the shikimate pathway were shown to be activated; the levels of related metabolites, especially phenylpropionic acid, tyrosine, shikimic acid, significantly increased; and the levels of hydroxycinnamic acid (HCA) derivatives and anthocyanins were elevated. The genes encoding sucrose degradation, the tricarboxylic acid cycle, and glycolysis were significantly up-regulated, and the hexose content also increased by more than 20 times. The amount of glutamine sharply decreased, and the conversion of N to C source provided a large amount of carbon for tumor development, indicating that the fungus induces the shikimate pathway and the flavonoid pathway, and the HCA derivatives are involved in lignin biosynthesis. Anthocyanins are involved in a variety of biotic and abiotic stresses. Although *U. maydis* does not directly contact the anthocyanins located in vacuoles, anthocyanin accumulation may be an indirect stress response caused by this fungus [63]. 

#### 3.1.4. *Rhizoctonia solani*–Plant Interaction 

*R. solani* is a causative agent of sheath blight, which leads to huge economic losses every year. By using UPLC-QTOF-MS metabolomics analysis, the metabolic variation of *R. solani* in vegetative, differentiated, and undifferentiated mycelia was detected. The results identified that some metabolites may act as biomarkers for the developmental stages of *R. solani* AG-1-IA. In addition, this research also revealed the mechanisms of sclerotium formation and mycelium differentiation at the metabolic level [64]. Similar work has also used this method to reveal the infection mechanisms of *R. solani* [65]. 

On the other hand, metabolic profiling strategies were also used to determine the mechanisms of plant defense against *R. solani*, including those in rice, soybean, lettuce, and potato [66,67,68,69,70,71,72]. In *R. solani*-infected soybean, global metabolism regulation was monitored over a time period. A comprehensive metabolite library for soybean infected by *R. solani* was subsequently constructed and will be used for metabolite identification and biological interpretation [67]. The study of metabolic networks of soybean revealed that *R. solani* infection resulted in the mobilization of carbohydrates, disturbance of the amino acid pool, and activation of the isoflavonoid, α-linolenate, and phenylpropanoid biosynthetic pathways. These pathways exhibit antioxidant properties and bioactivity that can help the soybean to counterattack *R. solani* infection. Unraveling the biochemical mechanism by metabolomics during the *R. solani*–soybean interaction provides valuable insights for crop breeding.

#### 3.1.5. *Botrytis cinerea*–Plant Interaction 

*B. cinerea* is a necrotrophic fungus, which can cause gray mold, one of the most serious diseases for some fruits. *B. cinerea* often results in a large amount of fruit rotting during harvest, storage, or transportation, causing serious economic losses. Global metabolomic analyses of *B. cinerea*-infected strawberry, grape, tomato, and *Arabidopsis* have been performed [73,74,75,76,77,78]. In *B. cinerea*-infected strawberry, metabolic profiling identified candidate biomarkers in the early stage of disease development when symptoms were not visible, which is potentially important for early diagnosis of *B. cinerea* [74]. The global metabolite changes induced by *B. cinerea* infection in grape were also detected by ^1^NMR to detect significant changes in chemicals or metabolites. This study revealed that *B. cinerea* infection causes significant metabolic changes in grape berry, and at the same time, metabolites derived from the plant and *B. cinerea* were both identified [76].

#### 3.1.6. Other Fungal Pathogen–Plant Interactions

*S. sclerotiorum* is a predominately necrotrophic fungal pathogen with a broad host range. A multiomic approach combining RNA sequencing, GC-MS-based metabolomics, and chemical genomics was performed on the *S. sclerotiorum*-infected resistant and susceptible soybean cultivars. The results identified an increase in bioactive jasmonate JA-Ile ((+)-7-iso-jasmonoyl-L-isoleucine), which scavenges reactive oxygen species and reprograms the phenylpropanoid pathway to increase antifungal activities in the resistant soybean [79].

*Colletotrichum*, a class of hemibiotrophic fungal pathogens, is one of the most widespread and economically detrimental genera of plant pathogenic fungi. An untargeted LC-MS metabolomic strategy was performed to elucidate metabolome changes in the anthracnose-causing *C. sublineolum* [80,81]. The results demonstrated through chemometric modelling revealed a metabolic variation trajectory, comprising three distinct stages that metabolically describe the adaptation of the fungus to diminishing nutrients. Using an UHPLC-HDMS analytical platform, Tugizimana et al. investigated the metabolic alterations of three sorghum cultivars responding to *C. sublineolum*, which revealed key characteristics of the biochemical mechanism underlying *C. sublineolum*–sorghum interactions and provided valuable insights with potential applications in crop breeding.

In the wheat pathogen *Stagonospora nodorum*, by using GC-ESI-MS/MS, Tan et al. found that the concentrations of secondary metabolites of the *Sch1* mutants were more than 200 times higher than those of the wild strain, which lays a solid foundation for elucidating the function of the *Sch1* gene [82]. Lowe et al. used GC-MS to perform non-targeted analysis of related metabolites in the formation of *S. nodorum* spores and found that chitosan plays an important role in sporulation [83].

Similar studies have also been carried out on the following interactions: *Fusarium oxysporum*–chickpea [84,85], *Verticillium dahliae*–*Arabidopsis* [86,87], *Verticillium longisporum*–*Arabidopsis* [88], *Venturia inaequalis*–apple [89], *Alternaria solani*–wild tomato [88], *Alternaria brassicicola*–*Arabidopsis* [90], *Gymnosporangium asiaticum*–Rosaceae plants [91], *Cercospora beticola*–sugar beet [92], *Plectosphaerella cucumerina*–*Arabidopsis* [93], *Aspergillus oryzae*–soybean [94], *Penicillium digitatum*–citrus [95], *Zymoseptoria tritici*–wheat [96], and *Alternaria alternata*–jujube fruit [97]. These studies have shown that metabolomics can be used to characterize plant-infecting fungal pathogens to identify some metabolites related to the resistance and to clarify plant resistance. These methods can also be used to determine the fungi-related metabolic mechanism, which is then used in fungicide development.

#### 3.1.7. Integrating Multi-Omics Assisted Metabolomics Research of Fungal Pathogen–Plant Interactions

There is growing interest in linking metabolomics with other omics tools, including genomics, transcriptomics, proteomics, and microbiomics. The integrated multi-omics strategies, in turn, could contribute to the comprehensive biological understanding that metabolomics studies alone would otherwise not achieve. A number of studies have reported on the use of integrated multi-omics based metabolomics research in fungal pathogen–plant interactions [68]. 

An integrated transcriptomics and metabolomics approach was used to uncover the primary metabolism regulation of soybean in response to *Rhizoctonia* infection [68]. Transcriptomics and metabolomics data were analyzed individually and integrated through the bidirectional orthogonal projections to latent structures (O2PLS), in order to reveal possible links between the metabolome and transcriptome during the early and late infection stages of the *Rhizoctonia*–soybean interaction. This study showed that alcohol and its associated gene product ADH (alcohol dehydrogenase) may have important roles in soybean resistance to *R. solani*. This study provided novel insights into the biological correlations and identification of metabolites that can be used in soybean breeding. A similar strategy was also used to reveal genes resistant to *Fusarium* head blight (FHB) in wheat QTL-Fhb2 [98] and the changes in the primary metabolism in bread wheat in response to *F. graminearum* [99]. 

Through a strategy combining proteomics and metabolomics, Kumar et al. revealed the metabolic reprogramming of chickpea infected by *F. oxysporum* f.sp. *ciceri* (Foc) [84]. They used quantitative label-free proteomics and ^1^NMR-based metabolomics to detect the dynamics in root metabolism during compatible and incompatible interactions between chickpea and Foc. The results showed a differential expression of proteins and metabolites in the resistant chickpea compared with the susceptible ones infected by Foc. Overall, the observed modulations in the metabolic flux, as an outcome of several orchestrated molecular events, were shown to be determinant of the plant’s role in chickpea–Foc interactions. A similar strategy was also used to decipher the mechanisms by which wheat QTL (Fhb1) resists *F. graminearum* [100] and to uncover novel proteins potentially involved in defense mechanisms against *Sclerotinia* in tomato overexpressing oxalate decarboxylase [101].

Pandey et al. integrated genomics, proteomics, and metabolomics approaches in order to determine whether oxalic acid functions as a pathogenic factor in *Tilletia indica* [102]. The results demonstrated that integrated omics approaches can be used to identify pathogenicity/virulence factor(s) that would provide insights into pathogenic mechanisms of fungi, which is therefore effective for developing new disease management strategies.

## 4. Prospects and Challenges

Metabolomics is still under rapid development. In the past decade, with the rapid development of analytical techniques, significant progress in metabolomics research has been made in determining the interactions between phytopathogenic fungal pathogens and their hosts. Information obtained from the metabolomics data is important for uncovering fungal infection mechanisms and plant defense mechanisms, which could be helpful for finding new targets for fungicide development and finding useful resistant genes for crop breeding.

There are still great challenges for metabolomics research in plant pathogenic fungi. Firstly, metabolomics needs to be coordinated with research on plant pathogenic fungi. For example, there are a lack of standard methods for quenching and extracting metabolites. Regarding the methodological study of technology platform integration, the complexity of biological samples poses higher demands for the sensitivity, resolution, dynamic range, and throughput of analytical techniques. In addition, the structural analysis of metabolites is a key step and difficult issue in metabolomics research. At present, there are also a lack of databases like GC-MS to aid in qualitative analyses. In theory, LC-MS-NMR can provide better structural information, but it cannot be widely used due to its complicated instruments, cumbersome operation, lack of sensitivity, low throughput, and high cost. Issues such as the construction of well-established metabolomics databases and standardized metabolomics research steps for plant pathogenic fungi have received increasing attention.

Secondly, fungus–plant interactions are very complex cascade processes, both in fungus infection processes and in plant defense responses as well as in fungus–plant communications. The final plant manifestation of disease resistance or disease susceptibility depends on the characteristics of both the plants and fungi involved. Metabolomics can be used to identify the antifungal substances produced by fungi in plants, to understand the physiological and biochemical processes of plants, and to detect the changes in certain key metabolites over time. More research is required to fully and accurately evaluate interaction-related metabolites and determine their functions. At present, fungal metabolomics research only focuses on the metabolites themselves and ignores their sources. For example, glucose from host and microbial metabolism is chemically and structurally identical, but the biological significance and related metabolic pathways of these types of metabolism lead to different regulatory pathways of glucose from these sources. In addition, confirmatory studies of the identified key metabolites are urgently needed. The potential biomarkers and metabolic pathways revealed in the metabolomics studies also require validation by independent biological studies.

Thirdly, multi-level omics data using integrated high-throughput technology, such as transcriptomics, proteomics, microbiomics, and metabolomics can help to identify new metabolites and major metabolic pathways in fungal pathogen–plant interactions. Combinations at different levels, such as gene expression and regulation as well as protein synthesis and expression, can help to elucidate biological processes that control metabolite levels and further identify relevant biomarkers. This will facilitate the analysis of the molecular mechanisms of plant responses to pathogenic fungal stress at a holistic level and accelerate the pace of biological research and agricultural applications. However, scientific research on the multiple platforms that comprehensively utilize systems biology is still scarce. Extensive research is required to make full use of metabolomics in the study of plant pathogenic fungi and to promote the prevention and control of crop fungal diseases.

## Figures and Tables

**Table 1:** Databases for metabolomics.

NO	Name	Website Address
1	ECMDB: The *Escherichia coli* Metabolome Database	http://www.ecmdb.ca/
2	YMDB: The *Yeast* Metabolome Database	http://www.ymdb.ca/
3	HMP: The Human Microbiome Project	http://www.hmpdacc.org/
4	EcoCyc: Encyclopedia of *Escherichia coli* K-12 Genes and Metabolism	http://www.ecocyc.org/
5	NMD: National Microbiological Database	http://www.foodsafety.govt.nz/industry/general/nmd/
6	MNPD: Microbial Natural Products Database	http://naturalprod.ucsd.edu/
7	UMBBD: University of Minnesota Biocatalysis/Biodegradation Database	http://umbbd.ethz.ch/
8	BioCyc Pathway	http://biocyc.org/
9	HMDB: Human Metabolome Database	http://www.hmdb.ca/
10	KEGG: Kyoto Encyclopedia of Genes and Genomes	http://www.genome.jp/kegg/
11	HumanCyc	http://bicyc.org
12	ARM	http://www.metabolome.jp
13	Lipidomics: Lipid Maps	http://www.lipidmaps.org/data/index.html
14	Lipidomics: SphinGOMAP	http://sphingomap.org/
15	Lipidomics: Lipid Bank	http://lipidbank.jp/
16	New drug and its metabolite database	http://www.ualberta.ca/_gjones/mslib.htm
17	ChemSpider Beta	http://www.chemspider.com
18	METLIN	http://metlin.scripps.edu/
19	MetaCyc Encyclopedia of Metabolic Pathways	http://metacyc.org/
20	PubChem Compound	http://www.pubmed.gov
21	SYSTOMONAS genome Database	http://systomonas.tu-bs.de/
22	PathDB: Pathogen Database	http://www.ncgr.org/pathdb/
23	NIST: National Institute of Standards and Technology	http://www.NIST.gov/srd/

**Table 2:** Recent metabolomics studies in fungal pathogen–plant interactions.

Fungal Pathogen	Plant Host	Platform	Year [Ref]
*Fusarium graminearum*	wheat	AP-SMALDI-MS	2018 [103]
	wheat	LC-ESI-LTQ-Orbitrap	2014 [104]
	barley	UHPLC-MS/MS	2014 [52]; 2011 [12]
	*Arabidopsis*	^1^H NMR	2018 [44]
	barley	LC-ESI-LTQ-Orbitrap	2012 [105]; 2010 [55]
*Fusarium oxysporum*	chickpea	^1^H NMR	2016 [84]
	chickpea	UHPLC-ESI-MS/MS	2015 [85]
*Fusarium tucumaniae*	soybean	GC-MS	2015 [106]
*Magnaporthe oryzae*	barley and rice	GC-MS	2009 [62]
	rice	^1^H NMR, LC-MS and GC-MS	2011 [58]
	rice	LC-MS and ^1^H NMR	2016 [61]
*Ustilago maydis*	maize	LC-MS	2008 [63]
			
			
			
*Rhizoctonia solani*	rice	UPLC-QTOF-MS	2017 [64]; 2018 [65]
	rice	^1^H NMR and LC-MS	2019 [72]
	rice	GC-MS and CE/TOF-MS	2017 [69]; 2016 [71]
	soybean	GC-MS	2014 [67]
	soybean	^1^H NMR	2017 [68]
	lettuce	GC-MS	2019 [66]
	potato	FT-ICR/MS and GC-EI/MS	2012 [70]
*Botrytis cinerea*	tomato	LC-MS and GC-MS	2015 [73]
	strawberry	GC-MS	2019 [74]
	*Arabidopsis*	DI-MS	2011 [75]
	grape	GC-MS	2017 [77]; 2015 [78]
	grape	^1^H NMR	2012 [76];
*Sclerotinia sclerotiorum*	common bean	UPLC-MS and GC-MS	2018 [79]
	tomato	UPLC-QTOF-MS/MS	2016 [101]
	soybean	GC-MS	2019 [107]
*Colletotrichum lupini*	lupin	LC-MS and GC-MS	2013 [108]
*Colletotrichum sublineolum*	sorghum	LC-ESI-QTOF-MS	2019 [80]
	sorghum	UHPLC-QTOF-MS	2019 [81]
*Verticillium dahliae*	*Arabidopsis*	GC-MS and LC-ESI-MS/MS	2015 [86]
	*Arabidopsis*	^1^H NMR	2018 [87]
*Verticillium longisporum*	*Arabidopsis*	UHPLC-QTOF-MS	2014 [89]
*Venturia inaequalis*	apple	GC-MS	2018 [88]
*Alternaria solani*	wild tomato	UPLC-QTOF-MS/LC-MS	2017 [90]
*Alternaria brassicicola*	*Arabidopsis*	GC-MS	2012 [109]
*Gymnosporangium asiaticum*	Rosaceae plants	GC-MS	2016 [91]
*Cercospora beticola*	sugar beet	(U)HPLC-UV-ESI-MS	2016 [92]
*Plectosphaerella cucumerina*	*Arabidopsis*	UPLC-QTOF-MS/MS	2016 [93]
*Aspergillus oryzae*	soybean	LC-ESI-MS and GC-TOF-MS	2014 [94]
*Penicillium digitatum*	citrus	GC–MS	2018 [95]
*Zymoseptoria tritici*	wheat	UHLC-MS/MS and GC-MS	2015 [96]
*Stagonospora nodorum*	wheat	GC-MS and ESI-MS/MS	2009 [82]
*Alternaria alternata*	jujube fruit	UPLC-QTOF-MS/MS	2019 [97]
