# Erratum: Chen, F. et al. Advances of Metabolomics in Fungal Pathogen–Plant Interactions. *Metabolites* 2019, *9*, 169

**DOI:** 10.3390/metabo9090175

**Published:** 2019-09-04

**Authors:** Fangfang Chen, Ruijing Ma, Xiao-Lin Chen

**Affiliations:** 1 State Key Laboratory of Biocatalysis and Enzyme Engineering, Hubei Collaborative Innovation Center for Green Transformation of Bio-Resources, School of Life Sciences, Hubei University, Wuhan 430062, China; 2 The Provincial Key Laboratory of Plant Pathology of Hubei Province, College of Plant Science and Technology, Huazhong Agricultural University, Wuhan 430070, China

The authors wish to make the following corrections to this paper [1].

In Page 10

On the other hand, metabolic profiling strategies were also used to determine the mechanisms of plant defense against R. solani, including those in rice, soybean, lettuce, and potato [66–72].

should be replaced with

On the other hand, metabolic profiling strategies were also used to determine the mechanisms of plant defense against R. solani, including those in rice, wheat, barley, soybean, lettuce, and potato [66–72].

In Table 2

For the fungal pathogen *Rhizoctonia solani*, the Plant Host corresponding to Ref [72] is not ‘rice’, but should be ‘wheat and barley’.

The authors would like to apologize for any inconvenience caused to the readers by these unintentional mistakes.
